# Nilotinib‐Induced Retinal Artery Thrombosis in a Patient With Chronic Myeloid Leukaemia: A Rare Case

**DOI:** 10.1002/cnr2.70501

**Published:** 2026-02-17

**Authors:** Vahid Mohammadkarimi, Abolfazl Khalafi‐Nezhad, Bijan Najafi, Nazanin Alemzadeh, Zahra Ghanbarinasab, Reza Vahid

**Affiliations:** ^1^ Hematology Research Center, Department of Hematology, Medical Oncology and Stem Cell Transplantation Shiraz University of Medical Sciences Shiraz Iran; ^2^ Department of Internal Medicine, School of Medicine Shiraz University of Medical Sciences Shiraz Iran

**Keywords:** chronic myeloid leukemia, nilotinib, retinal artery thrombosis

## Abstract

**Background:**

Tyrosine kinase inhibitors (TKIs) used in chronic myeloid leukemia (CML) are associated with a spectrum of cardiovascular toxicities, including hypertension, atrial fibrillation, impaired cardiac function, heart failure, and arterio‐occlusive events. Among these, nilotinib has been particularly linked to peripheral arterial disease and thromboembolic complications.

**Case:**

We report the case of a 53‐year‐old male patient who initially presented with fatigue, malaise, and mild left upper quadrant abdominal pain. Bone marrow aspiration confirmed CML with the presence of the *BCR::ABL1* fusion gene. The patient was started on nilotinib therapy. After 8 months of treatment, he developed unilateral blurred vision and sixth nerve palsy, which were attributed to a retinal arterial occlusion. No prior cardiovascular risk factors were documented. Nilotinib therapy was discontinued, and the patient was switched to dasatinib, leading to gradual improvement of visual symptoms.

**Conclusion:**

This case underlines the importance of considering rare but severe arterio‐occlusive complications, such as retinal artery thrombosis, among the cardiovascular toxicities of nilotinib. Careful cardiovascular monitoring and early recognition of such events are crucial to optimize patient safety and treatment outcomes in CML management.

## 1 Introduction

Chronic myeloid leukemia (CML) is a clonal myeloproliferative disorder of the hematopoietic stem cells, defined by the presence of the *BCR::ABL1* fusion gene resulting from the reciprocal translocation t(9;22)(q34;q11), also known as the Philadelphia chromosome. This fusion gene encodes a constitutively active tyrosine kinase that drives uncontrolled proliferation of myeloid cells and provides malignant cells with a survival advantage over normal hematopoiesis [1]. The discovery of tyrosine kinase inhibitors (TKIs) revolutionized the management of CML, transforming a once fatal hematological malignancy into a chronic, manageable condition with survival rates approaching those of the general population.

Currently, several TKIs, including imatinib, dasatinib, nilotinib, and bosutinib, are approved as first‐line options for CML [2]. While these drugs have dramatically improved long‐term outcomes, their benefits come with the cost of chronic toxicities, given that most patients require lifelong therapy [3]. Side effects of TKIs are diverse, affecting multiple organ systems, but cardiovascular toxicity has emerged as one of the most clinically significant complications. Reported cardiovascular adverse events include hypertension, atrial fibrillation, heart failure, impaired left ventricular function, and most importantly, arterio‐occlusive events [3, 4].

Within the class of second‐generation TKIs, nilotinib has been repeatedly linked to an increased incidence of vascular events. Although the precise mechanisms remain unclear, proposed contributors include impaired endothelial cell proliferation and migration, as well as associated metabolic alterations [5].

Although most documented vascular events linked to nilotinib involve peripheral or coronary arteries—such as Peripheral Arterial Occlusive Disease (PAOD), myocardial infarction, and cerebrovascular events—reports of ocular vascular complications (for instance, retinal arterial occlusion) are absent in the literature [6, 7]. Retinal arterial occlusion would be a vision‐threatening and life‐impacting event, yet to date no cases have been described in association with nilotinib—suggesting that either these events are exceptionally rare or underrecognized. Detailed case reports, if they emerge, would be invaluable for raising clinician awareness and refining our understanding of the TKI vascular risk profile.

Here, we describe a rare case of retinal artery thrombosis in a patient with CML treated with nilotinib, emphasizing the need for vigilant cardiovascular monitoring, early recognition of atypical vascular events, and prompt adjustment of therapy to optimize both oncologic and overall patient outcomes.

## 2 Case Presentation

### 2.1 Patient Information

53‐year‐old previously healthy man presented in 2023 with symptoms of fatigue, malaise, and mild left upper quadrant abdominal pain. He was admitted to the Hematology Department of Fars University of Medical Sciences in Shiraz, Iran, and further investigations were performed.

### 2.2 Clinical Findings and Diagnosis

On physical examination, moderate splenomegaly was noted, palpable 4 cm below the costal margin. No hepatomegaly, lymphadenopathy, or peripheral edema was detected.

Laboratory investigations revealed marked leukocytosis (WBC 151.2 × 10^9^/L), hemoglobin (11.7 g/dL) and platelet count (260 × 10^9^/L). Peripheral smear demonstrated left‐shifted granulopoiesis with neutrophilic predominance and the presence of bands, myelocytes, metamyelocytes, and occasional promyelocytes. Bone marrow aspiration showed a mildly hypercellular marrow with myeloid hyperplasia and no increase in blasts.

Molecular analysis by PCR confirmed the presence of the *BCR::ABL1* fusion transcript (t(9;22), p210, b3a2 isoform). Based on a Sokal score of 0.8, the patient was categorized as intermediate risk and diagnosed with chronic‐phase (CML).

**Table:** 

Complete blood count	
WBC	151.2 × 10^3^/L
Hemoglobin	11.7 g/dL
Platelets	260 × 10^3^/L
Fusion Transcripts	
BCR::ABL1	b3a2
Relative risk	
Sokal	Intermediate

### 2.3 Therapeutic Intervention

The patient was initiated on nilotinib 300 mg twice daily as first‐line therapy. At baseline, cardiovascular assessment, fasting blood glucose, and lipid profile were normal, and serial monitoring during therapy revealed no significant abnormalities. Importantly, the patient had no pre‐existing cardiovascular risk factors prior to initiation of nilotinib.

### 2.4 Adverse Event and Management

After 8 months of treatment, the patient developed sudden‐onset blurred vision in the left eye accompanied by diplopia due to sixth cranial nerve palsy. He had no associated systemic symptoms such as chest pain or claudication. Ophthalmological examination revealed reduced visual acuity, and fundus fluorescein angiography demonstrated retinal artery thrombosis of the left eye.

In the absence of conventional cardiovascular risk factors or alternative causes, the event was attributed to nilotinib‐associated arterio‐occlusive complications. Nilotinib was discontinued immediately. The patient received supportive ophthalmic care under specialist supervision, and dasatinib was initiated as an alternative tyrosine kinase inhibitor. Aspirin 80 mg daily was administered continuously throughout the course of therapy.

### 2.5 Follow‐Up and Outcome

The patient initially achieved complete hematologic response within the first month of therapy. After switching to dasatinib, his ocular symptoms gradually improved, and complete visual recovery was documented within several weeks.

On subsequent follow‐up, the patient has maintained hematologic remission on dasatinib with no evidence of recurrent vascular complications.

## 3 Discussion

The advent of tyrosine kinase inhibitors (TKIs) marked a paradigm shift in CML management. Imatinib, the first‐generation TKI, specifically targets the aberrant *BCR::ABL1* kinase, effectively suppressing leukemic proliferation and inducing durable remissions [8]. Subsequent development of second‐generation TKIs, including dasatinib, nilotinib, and bosutinib, provided more potent inhibition, particularly in patients resistant or intolerant to imatinib, and allowed for deeper molecular responses. These agents not only improve overall survival but also offer opportunities for treatment individualization based on disease risk, patient comorbidities, and anticipated adverse effect profiles [9].

Despite these advances, the use of TKIs is not without risk. While generally well‐tolerated, second‐generation TKIs, particularly nilotinib, have been associated with an increased incidence of vascular complications. Peripheral arterial occlusive disease, cerebrovascular events, and coronary artery disease are the most frequently reported adverse events [10].

Nazanin Aghel and Jeffrey Howard Lipton have reviewed the cardiovascular consequences of TKI in patients with CML. Their analysis indicates that nilotinib use is associated with an increased risk of cardiovascular complications, particularly acute coronary syndromes, ischemic stroke, and peripheral arterial occlusive disease, with the greatest risk observed at a dose of 400 mg twice daily. Moreover, nilotinib has been shown to negatively influence metabolic parameters, frequently resulting in hyperglycemia and dyslipidemia [11].

According to findings from a comparative meta‐analysis, initiation of therapy with nilotinib is associated with a greater likelihood of cardiovascular complications than treatment with imatinib [12]. The cardiotoxic effects of nilotinib are believed to arise primarily from promotion of atherosclerotic processes through inflammatory activity and from disturbances in glucose and lipid metabolism.

Importantly, dyslipidemia may improve after discontinuation of the drug. Proposed mechanisms include endothelial dysfunction, upregulation of adhesion molecules such as ICAM‐1, VCAM‐1, and E‐selectin, metabolic disturbances including dyslipidemia and hyperglycemia, and enhanced platelet activation. Notably, these vascular complications may arise even in patients without traditional cardiovascular risk factors, highlighting the importance of vigilant monitoring [13, 14].

Ocular vascular events, such as retinal artery occlusion, remain exceedingly rare but can have severe consequences, including permanent visual impairment. In our patient, nilotinib therapy at a dose of 300 mg twice daily initially achieved hematologic remission. However, 8 months into treatment, the patient experienced sudden unilateral blurred vision accompanied by sixth nerve palsy. Fundus fluorescein angiography confirmed thrombosis of the retinal artery in the affected eye. The absence of other contributory cardiovascular risk factors suggested a direct association with nilotinib therapy. Discontinuation of nilotinib and transition to dasatinib led to gradual resolution of visual symptoms.

This case underscores several critical clinical lessons. First, nilotinib‐induced arterio‐occlusive events are not confined to large peripheral or coronary arteries; microvascular territories, including the retinal circulation, can be affected. Second, clinicians should maintain a high index of suspicion for retinal arterial occlusion in patients presenting with visual disturbances during nilotinib therapy. Third, early intervention, such as switching to an alternative TKI, can allow continued disease control while mitigating further vascular risk. Finally, patient education regarding potential cardiovascular and ocular complications is essential to ensure timely reporting of new symptoms, facilitating early diagnosis and management.

In conclusion, nilotinib remains a highly effective therapy for CML but carries a clinically significant risk of vascular adverse events. Awareness of rare complications, such as retinal artery thrombosis, is critical to ensure prompt recognition and intervention, safeguard organ function, and optimize overall patient outcomes. Systematic cardiovascular and ocular monitoring should be considered as part of a comprehensive approach to patient care in CML.

## 4 Conclusion

This case report underscores the importance of vigilant monitoring for rare but serious arterio‐occlusive complications, such as retinal artery thrombosis, in patients with CML receiving nilotinib therapy. Clinicians should carefully balance the substantial therapeutic benefits of nilotinib against its potential cardiovascular risks, even in patients without traditional risk factors. Early recognition of vascular events, proactive screening, and management of modifiable cardiovascular risk factors are essential. A multidisciplinary approach involving hematologists, cardiologists, and ophthalmologists can optimize patient safety and treatment outcomes. Ultimately, individualized monitoring and timely intervention are crucial to minimize adverse events while maintaining the efficacy of tyrosine kinase inhibitor therapy.

## Author Contributions

All authors contributed to the collection of data, manuscript development and final approval. **Vahid Mohammadkarimi:** methodology (equal), conceptualization (lead), project administration (equal), resources (equal), supervision (equal), validation (equal), supervision (equal). **Abolfazl Khalafi‐Nezhad:** methodology (equal), conceptualization (lead), supervision (equal). **Bijan Najafi:** resources (equal), project administration (equal), conceptualization (lead). **Nazanin Alemzadeh:** writing – original draft (lead), writing – review and editing (lead). **Zahra Ghanbarinasab:** data curation (equal), investigation (lead), project administration (equal). **Reza Vahid:** data curation (equal), investigation (lead), project administration (equal). All authors have read and agreed to the published version of the manuscript.

## Funding

The authors have nothing to report.

## Ethics Statement

The ethics committee of Shiraz University of Medical Science approved this study IR.SUMS.MED.REC.1404.571. In accordance with international ethical standards, the author(s) have obtained and securely retained written ethical permission.

## Consent

Written informed consent has been obtained from the patient to publish this paper.

## Conflicts of Interest

The authors declare no conflicts of interest.

## Data Availability

The data that support the findings of this study are available on request from the corresponding author. The data are not publicly available due to privacy or ethical restrictions.
